# Green Solvents as an Alternative to DMF in ZIF-90 Synthesis

**DOI:** 10.3390/molecules26061573

**Published:** 2021-03-12

**Authors:** Aljaž Škrjanc, Ciara Byrne, Nataša Zabukovec Logar

**Affiliations:** 1 Department of Inorganic Chemistry and Technology, National Institute of Chemistry, Hajdrihova 19, SI-1001 Ljubljana, Slovenia; aljaz.skrjanc@ki.si (A.Š.); ciara.byrne@ki.si (C.B.); 2 Graduate School, University of Nova Gorica, Vipavska 13, SI-5000 Nova Gorica, Slovenia

**Keywords:** ZIF-90, green synthesis, GVL, DMF free, particle size control, Cyrene, room temperature synthesis

## Abstract

The use of green solvents as an alternative to dimethylformamide (DMF) in the synthesis of zeolitic imidazolate framework-90 (ZIF-90) was investigated. Two biobased aprotic dipolar solvents Cyrene^TM^ and γ-valerolactone (GVL) proved to successfully replace DMF in the synthesis at room temperature with a high product yield. While the Cyrene^TM^—based product shows reduced porosity after activation, the use of GVL resulted in materials with preserved crystallinity and porosity after activation, without prior solvent exchange and a short treatment at 200 °C. The primary particles of 30 nm to 60 nm in all products further form agglomerates of different size and interparticle mesoporosity, depending on the type and molar ratios of solvents used.

## 1. Introduction

Zeolitic imidazolate frameworks (ZIFs) are a subclass of metal-organic frameworks (MOFs), which have shown promising results as functional materials for gas storage [1,2,3,4], separation [5], and catalysis [6,7] applications. From the wide array of known ZIFs, ZIF-8 and ZIF-90, built from Zn-ions that are connected through 2-methylimidazolate or 2-carboxaldehyde imidazolate linkers into three-dimensional framework structures, make up more than half of all articles on ZIFs in the last 10 years. Their potential use in large-scale applications as functional materials also poses the question of atom economy in their synthesis, as well as its environmental impact. In an effort to alleviate the impact of the ZIF-8 synthesis, the use of recycled mother liquors has been recently reported [8]. However, implementation of greener solvents is also becoming an option in reducing the environmental impact of ZIF synthesis. While ZIF-8 like a number of other MOFs [9], has already been synthesised solvothermally in pure methanol [10] and even in water-based systems [11]. Additional ecofriendly methods such as electrochemical synthesis [12], mechanochemically [13], and even solvent and additive free from zinc oxide [14] have also been used for ZIF-8 synthesis. While ZIF-8 has many green alternative synthesis methods, ZIF-90 synthesis still largely relies on *N,N*-dimethylformamide (DMF) as a solvent [3,4,5,15,16,17,18].

The first step towards reducing the use of DMF was already taken in 2012 with introduction of the DMF/MeOH solvent system for ZIF-90 synthesis [19]. A search of articles on Web of Science using the keywords “green synthesis” and “ZIF-90” only finds seven publications at the time of writing this article, with three of them claiming a green synthetic procedure standing out more than the others [16,17,18]. All three articles use pure DMF as a solvent; Duan et al. [16,18] additionally uses *N, N*-diethylethanolamine while Zhang et al. [17] uses *N*,*N*,*N*′,*N*′- tetramethylethylenediamine. The paper [17] also reports the formation of spherical particles around 100 nm in diameter, which later form agglomerates with detectable mesoporosity depending on the amine content.

The only synthesis of ZIF-90 that applied the most principles of green chemistry was reported in 2013 with the introduction of a water-based synthesis procedure [20]. While water is the ideal green solvent, the procedure requires the addition of polymer polyvinylpyrrolidone (PVP). Additionally, the introduction of the water/alcohol/PVP system showed an interesting trend in the final solvent system composition-depended particle size, with the smallest particles being approximately 250 nm in diameter. While the synthesis looks promising as a green alternative, the lower Brunauer–Emmett–Teller (BET) of the water synthesised ZIF-90 makes the DMF-based synthesis route still slightly more appealing.

When looking for DMF alternatives for solvothermal synthesis, this study focused on dipolar aprotic solvents. Research into possible organic solvent replacements, which have already been previously used for MOF synthesis [9,21,22,23], led to two possible candidates: dihydrolevoglucosenone [24] with commercial name Cyrene^TM^, and gamma-valerolactone (GVL) [25,26]. Other possible new alternative solvents that could potentially be used were DMSO [23,27], TamiSolve^®^ [28], and methyl 5-(dimethylamino)-2-methyl-5-oxo- pentanoate [29]. However, due to no prior use for MOF synthesis, we decided to focus on GVL and Cyrene^TM^ as possible solvent alternatives.

Cyrene^TM^, is one of the more commonly known biobased alternative dipolar aprotic solvents. It is derived from cellulose via a 2-step catalytic reaction [24] with levoglucosenone as an intermediate product. Its boiling point is 203 °C [18]. The solvent has already been used in the synthesis of other MOFs, i.e., HKUST-1, UiO-66, Co-MOF-74, and Zn2(BDC)2(DABCO) [24]. The attempt to use it in the solvothermal synthesis of ZIF-8, reported in the same paper, resulted in problems with the aldol condensation product of Cyrene^TM^ after solvothermal synthesis at higher temperatures, which was difficult to fully remove and as such was a large impurity in the final product. 

GVL is also a popular bioderived solvent. It is produced from cellulose via the levulinic acid intermediate, with an alternative synthesis path via furfural from hemicellulose [22]. The solvent has previously been used successfully as the reaction media in catalytic C2–H arylation by using a series of Pd-loaded Zr-, Al-, Cu-, and Cr-based MOFs, where the stability of MOFs in GLV was assured [26]. GVL is praised for its biodegradability and nontoxicity, as well as its low melting point (−31 °C) and high boiling point (207 °C), which makes it a safe solvent for use on a larger scale [23]. Its high thermal stability [25] in the absence of an appropriate catalyst (150 °C up to several weeks) was also of note for pure thermal activation applications. It can be used safely in the presence of water at up to 60 °C with no noticeable decomposition, which makes it a good solvent for room temperature synthesis. Its decomposition in the presence of water reaches equilibrium after several days at 100 °C [25].

Both solvents are unstable in the presence of strong acids, with Cyrene^TM^ also being liable to strong oxidizing and reducing agents [23].

As a result of this, the aim of this paper is therefore to successfully synthesise ZIF-90 using GVL and Cyrene^TM^. Additionally, due to the proposed synthesis which requires no heating, the aldol condensation product is expected not to pose an issue during synthesis with Cyrene^TM^. Our study has shown that the synthesis of ZIF-90 can be carried out with a high yield, comparable to the DMF/MeOH-based synthesis, at room temperature, and without additional reagents, while using environmentally friendly solvents.

## 2. Results

### 2.1. XRD, SEM, and TG Analyses of As-Synthesised ZIF-90

**Figure 1:**
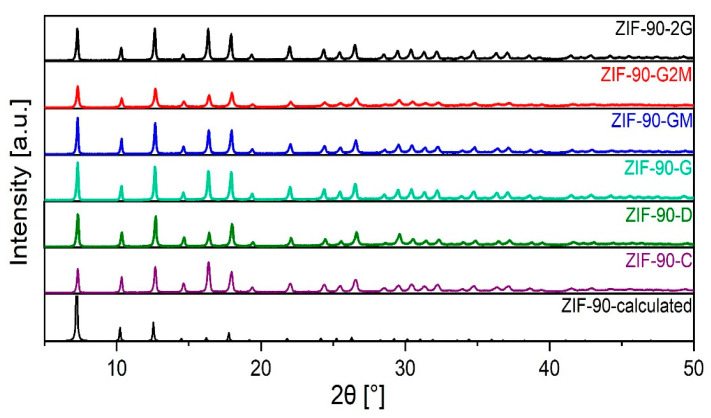
Powder X-ray diffraction data (PXRD) of synthesised ZIF-90.

The yields of the as-synthesised ZIF-90 (listed in Table 1) were similar for all solvent compositions. The PXRD analysis of all products shows that ZIF-90 structures were formed in all cases (Figure 1) with slight variation in intensities and peak width. No additional crystalline phases were detected.

**Figure 2:**
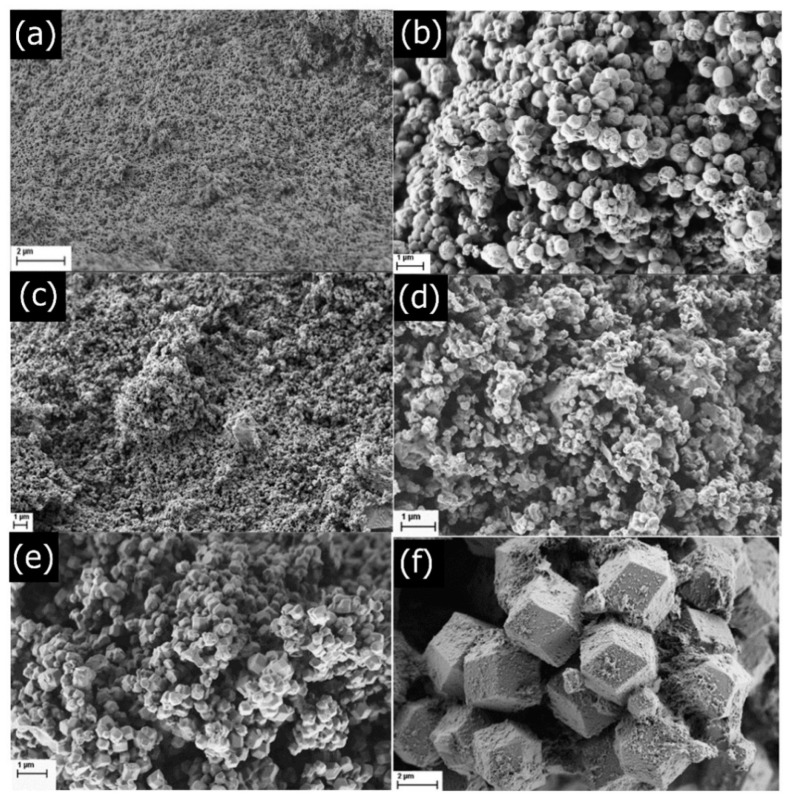
SEM images of the synthesised ZIF-90: (**a**) ZIF-90-D, (**b**) ZIF-90-C, (**c**) ZIF-90-2G, (**d**) ZIF-90-G, (**e**) ZIF-90-GM and (**f**) ZIF-90-G2M.

The samples were further investigated by SEM imaging of ZIF-90-D, ZIF-90-C, ZIF-90-G, ZIF-90-2G, ZIF-90-GM, and ZIF-90-G2M (Figure 2). Again, all products appeared to be in the pure phase with some traces of unreacted materials. Larger discernible particles were only seen in the SEM images of ZIF-90-G2M. The particle size estimated from SEM for ZIF-90-G2M was approximately 3 µm with other samples having particles with diameters in the range of 200 to 500 nm. However, since the width of diffraction maxima in PXRD patterns did not vary much for all products, further determination of particle size by using the Sherrer equation based on the collected XRD data was completed. The calculations revealed a nanosized particle distribution from 31 to 55 nm for the analysed products, i.e., calculated crystallite sizes were estimated to 55 nm for ZIF-90-D, 35 nm for ZIF-90-C, 53 nm for ZIF-90-G and ZIF-90-2G, 50 nm for ZIF-90-GM, and 31 nm for ZIF-90-G2M.

These results clearly indicate that the primary particles in all products are nanosized crystallites, which are further arranged in aggregates of different shapes and sizes. As all samples were synthesised with the GLV/MeOH ratio as the only variable in the synthesis, we could anticipate the ratio of GLV to methanol has an impact on particle agglomeration. This analysis showed that increasing the amount of methanol in syntheses resulted in larger agglomerates, from the smallest of ZIF-90-2G (<200 nm), ZIF-90-G (300 nm), ZIF-90-GM (500 nm), and finally the largest ZIF-90-G2M (3000 nm) agglomerates.

### 2.2. TGA and Activation

**Figure 3:**
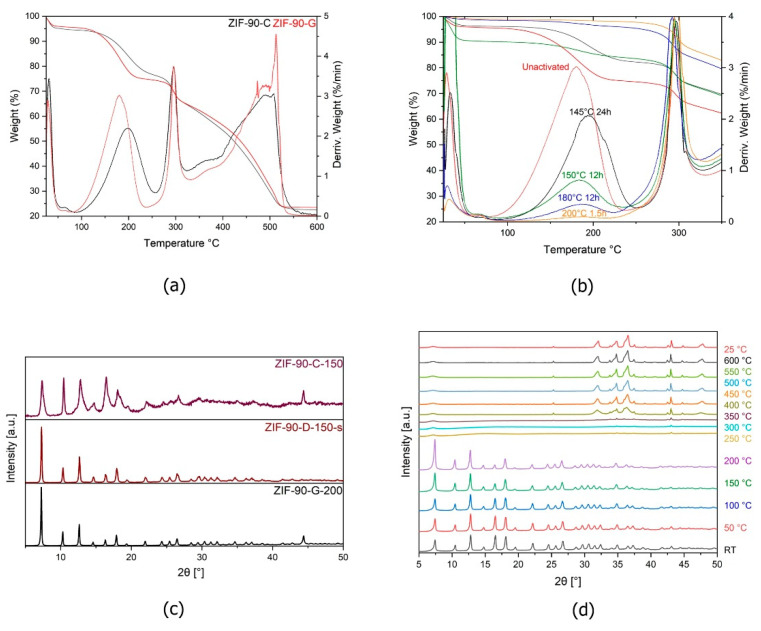
(**a**) TGA of ZIF-90-G and ZIF-90-C; (**b**) TGA of ZIF-90-G at different temperatures and times. For clarity, only the solvent peak region is shown; (**c**) PXRD of ZIF-90-C activated at 150, ZIF-90-D with classic solvent exchange activation and ZIF-90-G activated at 200 °C for 1.5 h; (**d**) High T PXRD for ZIF-90-G.

The TGA analysis was first performed for as-synthesised ZIF-90-G and ZIF-90-C. The first two prominent mass losses in the temperature range from 25 °C to 200 °C were attributed to solvent removal from pores of as-synthesised materials, with the first being around 30 °C (attributed to methanol) and the second being at 180 °C for GVL and 200 °C for Cyrene synthesised ZIFs (Figure 3a). The slightly delayed mass loss for ZIF-90-C compared to ZIF-90-G is contrary to what was expected, as Cyrene has a slightly lower boiling point than GVL [23]. However, it may be explained by the difference in size of the solvent molecules, as Cyrene is bulkier than GVL.

Following the TGA results, sample activation, i.e., removal of solvent from the pores, was carried out at different temperatures without prior solvent exchange. When doing preliminary thermal activation analysis for ZIF-90-C samples, PXRD showed structural degradation occurred at 150 °C (Figure 3c,d), despite the fact that TGA results anticipated structure stability up to higher temperatures. On the other hand, activation experiments for GVL synthesised ZIF-90-G (Figure 3b) at different temperatures (145 °C, 150 °C 180 °C, 200 °C) with varying times (1.5–24 h) resulted in well-preserved crystallinity of samples. According to the TGA results, activation of materials was only completed at 200 °C after only 1.5 h of heating. At lower T, the activation was only partial (TG still showed mass loss at 180 °C) even with prolonged time of up to 24 h in the vacuum oven. The results confirmed that temperature is a crucial parameter when it comes to pure thermal activation attempts, as small changes in temperature showed larger differences in solvent removal, compared to changes in time of activation. Better solvent removal could be explained with activation temperature nearing the boiling point of GVL when using pure thermal activation. Other samples were also analysed using TGA and XRD, with minor differences between them (Figure S1).

The changes in crystallinity after activation were checked by PXRD for ZIF-90-C-150 (150 accounts for activation at 150 °C), and for ZIF-90-G-200 (200 accounts for activation at 200 °C) (Figure 3c). In addition to these, the PXRD patterns were also collected for ZIF-90-D-150-s. No difference in crystallinity between ZIF-90-D-s and ZIF-90-G-200 was observed.

**Figure 4:**
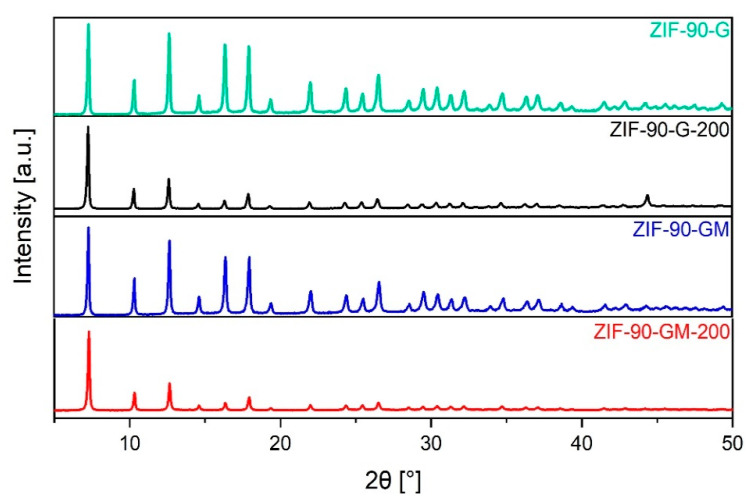
PXRD of ZIF-90-G and ZIF-90-GM before and after thermal burst activation at 200 °C.

Afterwards, the high temperature “thermal burst” activation at 200 °C for a short amount of time, i.e., 1.5 h, was successfully applied with all GVL synthesised ZIF-90. Despite the relatively high temperature of activation, the PXRD showed no obvious structural degradation (Figure 4 and Figure S2). Based on obtained results, including the highest crystallinity and similar agglomerate size, we selected thermally activated ZIF-90-G and ZIF-90-GM for further examination of textural properties.

### 2.3. N_2_ Adsorption Isotherm Measurements

**Figure 5:**
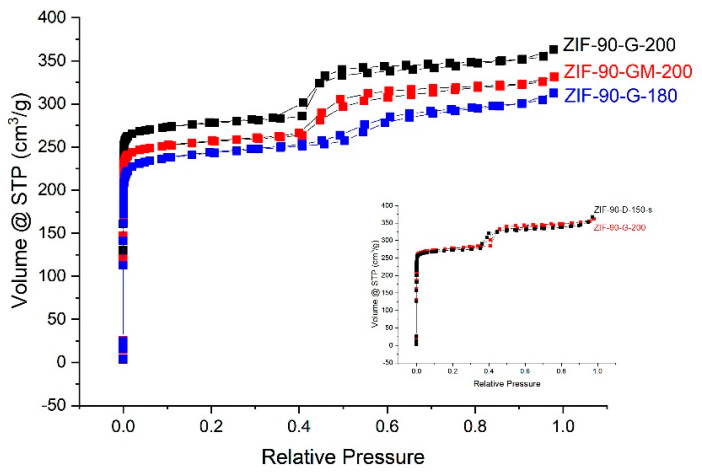
N_2_ adsorption isotherms for fully activated (ZIF-90-G-200, ZIF-90-GM-200), while the inset shows partially activated (ZIF-90-G-180) and reference (ZIF-90-D-150-s) samples.

**Figure 6:**
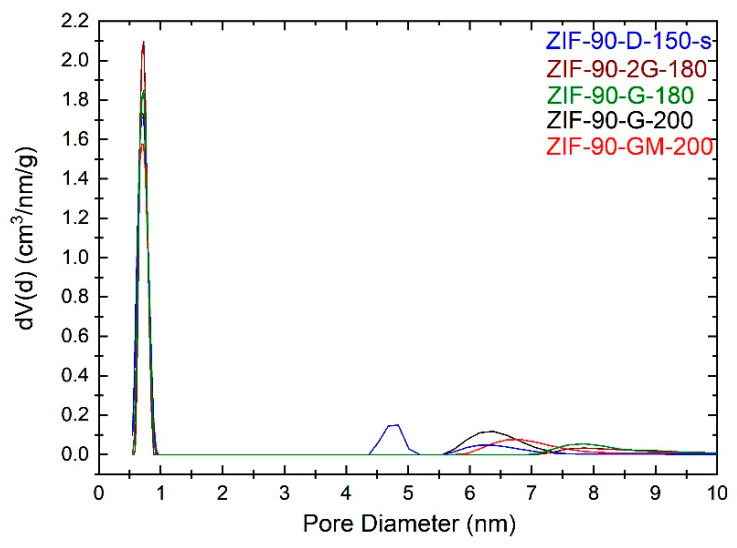
Pore size distribution graph calculated from nitrogen physisorption isotherms.

For the study of porosity, the focus was on ZIF-90 products prepared with GLV, as Cyrene-based products revealed a reduction of crystallinity after activation. It indicated the influence of solvent as well as the activation procedure on textural properties of the selected materials. The selected N_2_ adsorption isotherms are shown in Figure 5. The BET, total pore volume, and pore diameter were calculated using the built-in functions of the software (Table 2). Pore size distribution is presented in Figure 6.

The results for ZIF-90-G-180 and ZIF-90-G-200 samples confirm that lower activation temperature, i.e., 180 °C, results in lower BET values, compared to activation at 200 °C. Furthermore, by comparing BET values of ZIF-90-G-200 and ZIF-90-GM-200, activated under the same conditions, it can be seen that material prepared from mixed-solvent system (GLV:MeOH = 1:1) shows lower BET value than the pure GLV system. The comparison with ZIF-90-D-150-s, as a reference material, revealed that it has a BET value slightly lower than ZIF-90-G-200. The obtained BET values, listed in Table 2, were higher than some other BET values from the literature, i.e., 717.9 [4], 785 [20], and 766 [20] m^2^/g, yet still lower than the reported maximum of 1426 [5] m^2^/g for DMF synthesised ZIF-90 comparison with some additional BET values from literature is shown in Table S1.

The total pore volume and micropore volume of analysed materials varied slightly, which was attributed to differences in distribution of micro- and mesopores, as shown in Figure 6. The presence of mesopores is a consequence of interparticle porosity between the primary nanoparticles and the differences in micro-mesopores distribution a consequence of the size and degree of agglomeration of nanoparticles in the final products [30].

## 3. Discussion

Successful substitution of DMF for biomass-based solvents was achieved for the ZIF-90 synthesis procedure. During the syntheses, a difference in solubility was observed. The linker, 2-carboxaldehyde imidazole, was less soluble in the green solvents Cyrene and GLV and as such the synthesis time was elongated by 30 min to account for lower solubility. By partial substitution of GLV with MeOH, it was possible to achieve a complete dissolution of the linker at room temperature and without prior heating to facilitate linker dissolution, as required in the Brown et al. [19] procedure. The pH value of approximately 5, measured for both green-solvent-based syntheses, was compatible with green solvent requirements since both are instable in strongly acidic conditions [23]. In order to further reduce the environmental impact of ZIF-90 synthesis, the regeneration of solvents from the mother liquid after isolation could be applied. The most probable regeneration could be done with fractional distillation due to the large difference in boiling point of the two solvents, followed by the analysis of the composition of the mother liquid as well as stability of GVL after multiple cycles of recycling.

Analysis of the synthesised ZIF-90 samples showed slight differences in the size of primary particles and more pronounced differences in the size of agglomerates formed in different solvents. Literature on the reaction mixture impact on particle size of MOFs has shown solvent composition dependant particle size for ZIF-90 [20] and for Al-based MOF NH_2_-MIL-53(Al) [31], as well as an excess linker dependant agglomeration of particles in ZIF-8 system [32]. Additional studies showed that agglomeration could also be dependent on template (amines) to zinc ion ratio for ZIF-90 [17]. In the water/PVP/alcohol system a further correlation was drawn on mixed solvent viscosity on particle size (i.e., the type of alcohol used), with viscosity having an inverse correlation with particle size [20]. Solvothermally synthesised ZIF-90 particle size has even been shown to be mixing speed- and time-dependant prior to heating in autoclave [33]. However, the solvent composition dependant particle agglomeration of ZIF-90 has not been reported yet. Although we observed difference in particle size depending on the used solvent mixture, further research needs to be done to systematically evaluate the influence of time, temperature, and solvent composition ratio on particle/agglomerate size.

Furthermore, we successfully applied a modified activation procedure to the materials. Current activation techniques follow the route of exchanging the high boiling point dipolar aprotic solvent for a solvent with a low boiling point and high vapour pressure, most commonly methanol, by soaking the sample in it. This is not the best approach from the sustainability standpoint, as it requires the application of additional solvent. To that end, activation without prior solvent exchange was attempted. The pure thermal activation was tested for both green solvent systems. The first was tried at 150 °C overnight. The PXRD of the samples afterwards showed structural collapse in the case of ZIF-90-C. Cyrene^TM^ reportedly [24] forms aldol condensation products. PXRD analysis of as-synthesised ZIF-90-C samples showed no diffraction maxima that could be attributed to the aldol condensation product. Analysis of PXRD data after the activation attempt also could not confirm the presence of an aldol condensation product, but in this case it was mostly because of the overlapping and broad diffraction maxima, which hindered a conclusive phase determination. Due to the absence of aldol condensation impurity in as-synthesised samples it can be anticipated that classic solvent exchange could be potentially used. On the other hand, the structures obtained by using GLV as a solvent retained their crystallinity when following the described thermal activation. Furthermore, it was demonstrated that the “thermal burst”-activated products from the GLV-based synthesis show textural properties (BET, total pore volume and average pore diameter) comparable to ZIF-90-D-150-s, as a reference material (Table 2). The activation technique shows promise in that it is fast and as such could lead to better throughput of activated sample.

## 4. Materials and Methods

### 4.1. Materials

*N*,*N*-dimethylformamide (DMF, >99%), Methanol (MeOH, >99%), gamma-valerolactone (GVL, 99%), dihydrolevoglucosenone(Cyrene^TM^, 98.5%) and zinc acetate dihydrate (Zn(AcO)_2_.2H_2_O, 98%) were purchased from Sigma Aldrich (Darmstadt, Germany). 2*H*-imidazole carbaldehyde (HICA, 97%) was purchased from Fluorochem (Hadfield, UK). All chemicals were used without further purification.

### 4.2. ZIF-90 Synthesis

The synthetic procedure was based on an in-lab developed synthesis method for DMF synthesised ZIF-90, which was modified from Brown et al. [19].

The use of Cyrene^TM^ and GVL were examined in the synthesis procedure with a 1 to 1 volumetric substitution of DMF for green solvent. The largely different solubility of the imidazole linker in green solvents, compared to DMF, led to modification of the procedure in a way that skips the heating step in the linker solution preparation. To compensate for the solubility issue, the reaction time was extended from 30 min to 1 h. The system had a comparable yield to the DMF/MeOH procedure. GVL substitution had a further modification where we exchanged pure solvent for mixed solvent of MeOH/GVL with different molar ratios. During the hour-long synthesis, the pH was checked at the 10 min and 50 min marks using a pH strip. The pH was around 5 to 5.5 with no changes in the value at the beginning and the end of the reactions.

#### 4.2.1. ZIF-90-D Synthesis

Modified from Brown [19], 1.93 g (19 mmol) of HICA and 50 mL DMF (645 mmol) was added to a 100 mL beaker and stirred at 70 °C until a clear solution formed, then left to sit in the fume hood until it cooled to room temperature. Meanwhile 1.46 g (6.5 mmol) of Zn(AcO)_2_·2H_2_O was dissolved in 50 mL MeOH (1230 mmol) in a 150 mL beaker. When cooled, the HICA solution was slowly poured into the zinc solution and left to stir for 30 min. After 30 min, the formed precipitate was isolated by centrifuge 9000 rpm for 35 min. The precipitate was then suspended in MeOH, vacuum filtered and washed with MeOH again, then left to air-dry overnight.

#### 4.2.2. Green Solvent Based ZIF-90 Syntheses

In a 100 mL beaker, 1.93 g (19 mmol) of HICA was added to 50 mL linker solvent (Table 1). The mixture was left to stir at room temperature until fully suspended in the solvent. A separate solution of 1.46 g (6.5 mmol) of Zn(AcO)_2_·2H_2_O in 50 mL of metal precursor solvent (Table 1) was prepared in a 150 mL beaker. The HICA suspension was then slowly poured into the zinc solution and left to stir for 1 h. The product was isolated by centrifuge 9000 rpm for 35 min, washed with MeOH, and centrifuged again. The precipitate was left to air-dry overnight in the centrifuge bottle.

### 4.3. Activation of ZIFs

#### 4.3.1. Solvent Exchange Activation

The ZIF-90 products were transferred to glass vials and left to soak in methanol for 5 h with agitation by occasional shaking during the first few hours. The methanol was then poured off and the vials left to air-dry until visibly dry. The dried samples were then heated in a vacuum oven at 150 °C overnight.

#### 4.3.2. Thermal Burst Activation

Samples were transferred to glass vials while making sure to not overfill them. Attention was also given so that the layer at the bottom of the vial was not to too thick. The oven was then put under vacuum, heated up to 200 °C, and held at that temperature for 1.5 h.

### 4.4. Characterisation

Powder X-ray diffraction data (PXRD) were recorded on a PANanalytical X’Pert PRO high-resolution diffractometer using CuK_α1_ radiation (1.5406 Å) in the 2θ range from 5 to 50° (100 s per step 0.033° 2θ) with a fully opened X’Celerator detector. The diffractograms were analysed and the particle size calculated using the Sherrer equation with the HighScore Plus 4.9 program package (Malvern Panalytical B.V.).

Nitrogen physisorption isotherms were recorded at −196 °C using the Autosorb iQ3. Before the adsorption analysis, the samples were degassed under vacuum for 10 h at 150 °C. The Brunauer–Emmett–Teller (BET) specific surface area was calculated from adsorption data in the relative pressure range from 0.05 to 0.2. The total pore volume (V_total_) was calculated from the amount of N_2_ adsorbed at P/Po = 0.97 and micropore volume from t-plot (p/p_0_ = 0.15 − 0.3).

Thermogravimetric analysis (TGA) was performed on a TA Instruments Q5000. The measurements were carried out in a continuous airflow (25 mL/min air, 10 mL/min Ar), by heating samples from 25 °C to 650 °C at a rate of 10 °C/min.

Scanning electron microscope (SEM) images were taken using a Zeiss Supra 35 VP microscope with an electron high tension voltage of 1.00 kV and Aperture Size 30.00 μm.

## 5. Conclusions

This study shows that we have successfully substituted DMF, with green solvent alternatives (Cyrene^TM^ and GLV) in a synthesis procedure of ZIF-90, with a further introduction of “thermal burst” activation. From all of the investigated synthesis procedures, the synthesis of ZIF-90-G and ZIF-90-GM showed the most promise. The procedures use an environmentally unproblematic biomass derived solvent and cut the costs of activation by using no additional solvent. The synthesis also requires no additional base or polymer to facilitate precipitation. This lends itself as a promising alternative solvothermal synthesis method for ZIF-90 for further applications in sorption and other applications. Variation in GVL/MeOH ratio of the mixed solvent linker solution could allow for control of size of particles/agglomerates and associated mesoporosity of synthesised ZIF-90 but requires further investigation.

## Figures and Tables

**Table 1:** Solvent composition for the synthesised zeolitic imidazolate frameworks (ZIF)-90 samples.

Product	Linker Solvent	Metal Precursor Solvent
ZIF-90-D	DMF (645 mmol) *	MeOH (1250 mmol)
ZIF-90-C	Cyrene^TM^ (481 mmol)	MeOH (1250 mmol)
ZIF-90-G	GVL (525 mmol)	MeOH (1250 mmol)
ZIF-90-2G	GVL (263 mmol) + MeOH (625 mmol)	GVL (263 mmol) + MeOH (625 mmol)
ZIF-90-GM	GVL (263 mmol) + MeOH (625 mmol)	MeOH (1250 mmol)
ZIF-90-G2M	GVL (179 mmol) + MeOH (825 mmol)	MeOH (1250 mmol)

* Requires heating to 70 °C and then cooling to room temperature.

**Table 2:** Activation parameters, Calculated Brunauer–Emmett–Teller (BET), total pore volume, and average pore diameter for synthesised ZIFs.

ZIF	Activation	S_BET_^2^ (m^2^/g)	V_total_^3^ (cm^3^/g)	V_micro_^4^ (cm^3^/g)
ZIF-90-D-150-s	150 °C/12 h ^1^	1119	0.514	0.390
ZIF-90-2G-180	180 °C/12 h	929	0.420	0.392
ZIF-90-G-180	180 °C/12 h	977	0.437	0.331
ZIF-90-G-200	200 °C/1.5 h	1136	0.508	0.311
ZIF-90-GM-200	200 °C/1.5 h	1044	0.465	0.358

^1^ prior to heating soaked in MeOH, ^2^ S_BET_—Specific surface area was calculated by the BET method using adsorption data in the range of P/P_0_ = 0.05 − 0.2; ^3^ Vtotal—Total pore volume was calculated based on the nitrogen adsorption amount at p/p_0_ = 0.97, ^4^ t-plot method (p/p_0_ = 0.15 − 0.3).

## Data Availability

The data presented in this study is available on request from the corresponding author.

## Supplementary Materials

The following are available online, Figure S1: TGA of synthesised ZIFs before and after activation, XRD of ZIF-90-2G before and after activation, Table S1: Literature BET and particle size values for some synthesis methods.

## Sample Availability

Samples of the compounds are not available from the authors.
