# Supplementary material for: Green Solvents as an Alternative to DMF in ZIF-90 Synthesis
Source: Molecules. 2021 Mar 12;26(6):1573. doi: 10.3390/molecules26061573 (PMC8001175; doi:10.3390/molecules26061573)
Supplement: Supplementary file 1 [file molecules-26-01573-s001.pdf]

Supplementary Materials

Green Solvents as an Alternative to DMF in ZIF-90 Synthesis

Aljaž Škrjanc [1,2] , Ciara Byrne[1] and Nataša Zabukovec Logar [1,2,*]

1 Department of Inorganic Chemistry and Technology, National Institute of Chemistry, Hajdrihova 19, SI-1001 Ljubljana, Slovenia; aljaz.skrjanc@ki.si
2 University of Nova Gorica, Vipavska 13, SI-5000 Nova Gorica, Slovenia
* Correspondence: natasa.zabukovec@ki.si

S1. Literature BET and particle size data for ZIF-90

Table S1: Literature BET and particle size values for some synthesis methods

Solvent	S_{BET} [m²/g]	Particle size [nm]	Reference
DMF	1182	2000	**[1]**
DMF	1270	1430	**[2]**
H_2O	785	2130	**[2]**
H_2O/butanol	766	275	**[2]**
DMF	1106	55	**[3]**
DMF	1426	79	**[4]**
DMF	718	80 – 100	**[5]**
DMF	529	100*	**[6]**
DMF	394	70*	**[6]**
DMF	717	/	**[7]**

1. Liu, C.; Liu, Q.; Huang, A. A superhydrophobic zeolitic imidazolate framework (ZIF-90) with high steam stability for efficient recovery of bioalcohols. *Chem. Commun.* **2016**, *52*, 3400–3402, doi:10.1039/c5cc10171a.

2. Shieh, F.K.; Wang, S.C.; Leo, S.Y.; Wu, K.C.W. Water-based synthesis of zeolitic imidazolate framework-90 (ZIF-90) with a controllable particle size. *Chem. - A Eur. J.* **2013**, *19*, 11139–11142, doi:10.1002/chem.201301560.

3. Hua, D.; Ong, Y.K.; Wang, Y.; Yang, T.; Chung, T.S. ZIF-90/P84 mixed matrix membranes for pervaporation dehydration of isopropanol. *J. Memb. Sci.* **2014**, *453*, 155–167, doi:10.1016/j.memsci.2013.10.059.

4. Zhang, Q.; Luo, S.; Weidman, J.; Guo, R. Surface modification of ZIF-90 with triptycene for enhanced interfacial interaction in mixed-matrix membranes for gas separation. *J. Polym. Sci.* **2020**, *58*, 2675–2687, doi:10.1002/pol.20200123.

5. Ghahramaninezhad, M.; Mohajer, F.; Niknam Shahrak, M. Improved CO2 capture performances of ZIF-90 through sequential reduction and lithiation reactions to form a hard/hard structure. *Front. Chem. Sci. Eng.* **2020**, *14*, 425–435, doi:10.1007/s11705-019-1873-5.

6. Zhang, H.; Duan, C.; Li, F.; Yan, X.; Xi, H. Green and rapid synthesis of hierarchical porous zeolitic imidazolate frameworks for enhanced CO2 capture. *Inorganica Chim. Acta* **2018**, *482*, 358–363, doi:10.1016/j.ica.2018.06.034.

7. Akbari Beni, F.; Niknam Shahrak, M. Alkali metals-promoted capacity of ZIF-8 and ZIF-90 for carbon capturing: A molecular simulation study. *Polyhedron* **2020**, *178*, 114338, doi:10.1016/j.poly.2019.114338.

S2. TGA and XRD of synthesised ZIF-90

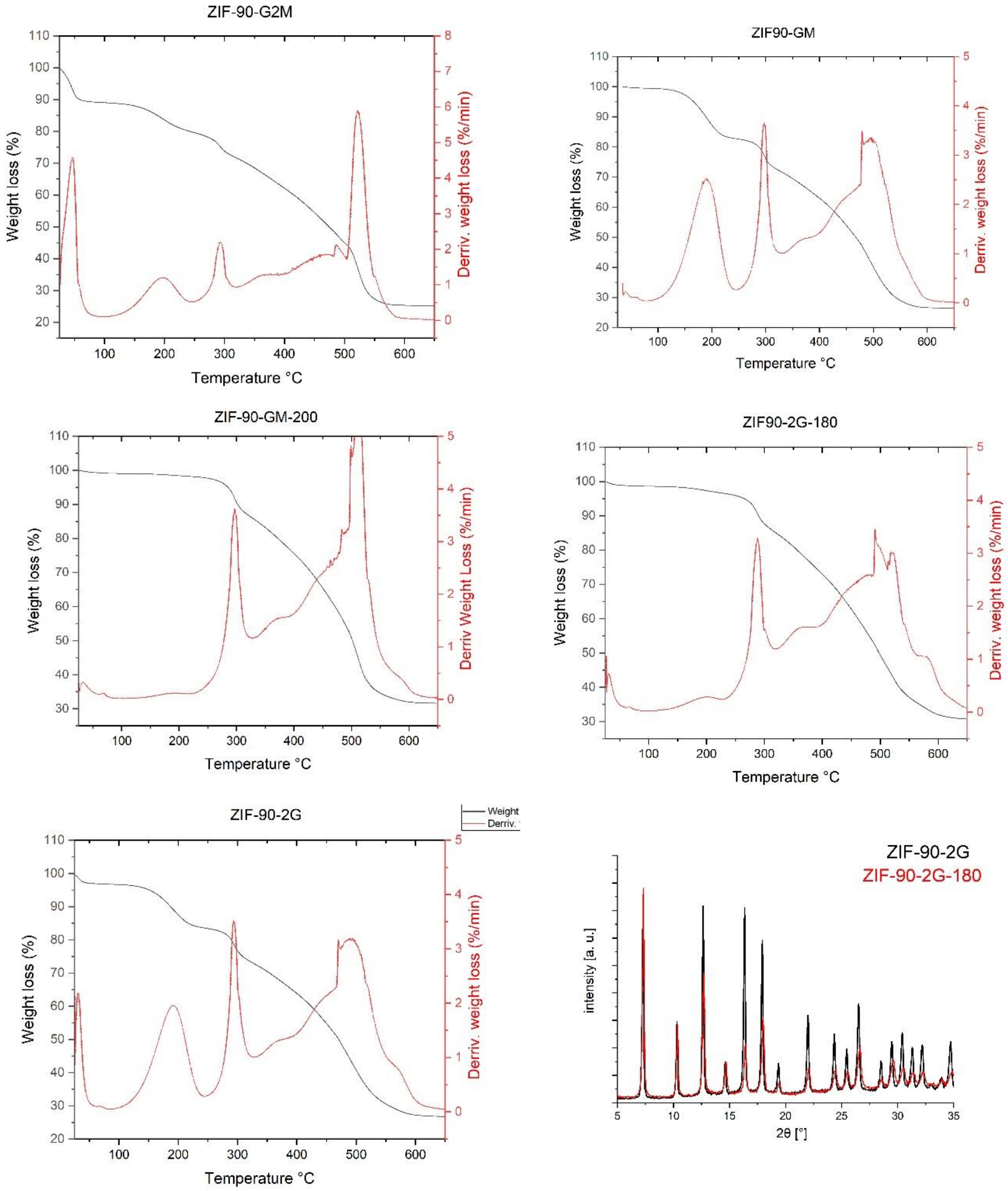

Figure S1 TGA of synthesised ZIFs before and after activation, XRD of ZIF-90-2G before and after activation
